# Intravenous versus epidural analgesia to reduce the incidence of gastrointestinal complications after elective pancreatoduodenectomy (the PAKMAN trial, DRKS 00007784): study protocol for a randomized controlled trial

**DOI:** 10.1186/s13063-016-1306-4

**Published:** 2016-04-11

**Authors:** Rosa Klotz, Stefan Hofer, Alexander Schellhaaß, Colette Dörr-Harim, Solveig Tenckhoff, Thomas Bruckner, Christina Klose, Markus K. Diener, Markus A. Weigand, Markus W. Büchler, Phillip Knebel

**Affiliations:** Department of General, Visceral and Transplantation Surgery, Heidelberg University Hospital, Im Neuenheimer Feld 110, 69120 Heidelberg, Germany; The Study Center of the German Surgical Society (SDGC), Heidelberg University Hospital, Im Neuenheimer Feld 130.3, 69120 Heidelberg, Germany; Department of Anesthesia, Heidelberg University Hospital, Im Neuenheimer Feld 110, 69120 Heidelberg, Germany; Department of Anesthesia, Intensive Care and Emergency Medicine, Red Cross Hospital Kassel, Hansteinstrasse 29, 34121 Kassel, Germany; Institute of Medical Biometry and Informatics, University of Heidelberg, Im Neuenheimer Feld 130.3, 69120 Heidelberg, Germany

**Keywords:** Epidural analgesia, Patient-controlled intravenous analgesia, Pancreatoduodenectomy, Postoperative pain management, Postoperative complication, Randomized controlled trial

## Abstract

**Background:**

Despite substantial improvements in surgical and anesthesiological practices leading to decreased mortality of less than 5 % at high-volume centers, pancreatic surgery is still associated with high morbidity rates of up to 50 %. Attention is increasingly directed toward the optimization of perioperative management to reduce complications and enhance postoperative recovery. Currently, two different strategies for postoperative pain management after pancreatoduodenectomy are being routinely used: patient-controlled intravenous analgesia and thoracic epidural analgesia. Evidence is lacking to assess which strategy entails fewer postoperative complications.

**Methods/design:**

The PAKMAN trial is designed as an adaptive, pragmatic, randomized, controlled, multicenter, open-label, superiority trial with two parallel study groups. A total of 370 patients scheduled for elective pancreatoduodenectomy will be randomized after giving written informed consent, and 278 patients are needed for analysis. Patients with chronic pancreatitis, severe chronic obstructive pulmonary disease (COPD), American Society of Anesthesiologists (ASA) physical status classification ≥ IV, or chronic pain syndrome will be excluded. The group A intervention includes intraoperative general anesthesia and postoperative patient-controlled intravenous analgesia; the group B intervention comprises combined intraoperative general anesthesia and epidural analgesia with postoperative epidural analgesia. The primary endpoint of this trial is a composite of the gastrointestinal complications (delayed gastric emptying, pancreatic fistula, biliary leak, gastrointestinal bleeding, and postoperative ileus) up to postoperative day 30. The aim is to investigate whether the frequency of gastrointestinal complications following pancreatoduodenectomy can be reduced by 15 % using postoperative, patient-controlled intravenous analgesia compared with epidural analgesia.

**Discussion:**

Several previous studies investigating the two different strategies for postoperative pain management have mainly focused on their effectiveness in pain control. However, the PAKMAN trial is the first to compare them with regard to their impact on the surgical endpoint “postoperative gastrointestinal complications” after pancreatoduodenectomy.

**Trial registration:**

German Clinical Trials Register, DRKS00007784

**Electronic supplementary material:**

The online version of this article (doi:10.1186/s13063-016-1306-4) contains supplementary material, which is available to authorized users.

## Background

Owing to significant improvements in surgical and anesthesiological practices, mortality in pancreatic surgery has been reduced to less than 5 % at high-volume centers. However, even specialized centers with highly standardized surgical techniques still report morbidity rates of up to 50 %, mainly due to gastrointestinal complications [1]. As one of the most important complications, postoperative pancreatic fistula can lead to further adverse effects such as erosion bleeding, intra-abdominal abscess, or sepsis [2]. With regard to patient outcome after pancreatoduodenectomy, the recently published RecoPanc trial (DRKS00000767) compared pancreatogastrostomy with pancreatojejunostomy and showed no significant difference in the rate of pancreatic fistula [3]. Along with the constant refinement of surgical technique during the past two decades, increasing attention is now being directed toward the optimization of perioperative management to reduce complications and enhance postoperative recovery.

The most common strategies for effective perioperative pain management in major abdominal surgery are patient-controlled intravenous opioid analgesia (IV-PCA) and epidural analgesia (EDA) [4]. A retrospective study of perioperative pain management strategies in pancreatic surgery published in 2008 [5] found that EDA with additional intravenous analgesia was associated with a lower occurrence of postoperative gastrointestinal and infectious complications than EDA alone. The aim of the trial described here is to verify this association in a multicenter RCT.

EDA is usually achieved by combined injection of local anesthetics and opioids into the epidural space. This inhibits neural transmission and induces analgesia. Additionally, the action of the sympathetic nervous system is lessened, and vasodilation is provoked. Major abdominal surgery frequently entails large volume shifts and significant blood losses. Some evidence exists that EDA provokes hemodynamic instability in this setting [6, 7]. Typically, vasopressors and large amounts of fluids are administered to counteract undesirable hypotension. These interventions may affect the healing of gastrointestinal anastomoses in a negative manner and possibly increase the incidence of complications such as anastomotic insufficiency. A prospective study identified hypotension during abdominal surgery as a significant risk factor for postoperative complications [8]. In a randomized controlled trial (RCT) of patients undergoing pancreatoduodenectomy, greater intraoperative fluid administration resulted in more pancreatic anastomotic complications [9]. Pratt et al. showed a significant decrease of 15 % in gastrointestinal complications for patients undergoing pancreatoduodenectomy with postoperative intravenous analgesia versus thoracic EDA [5].

Although thoracic EDA is effective in pain control, its possible adverse effects on postoperative recovery of patients after pancreatoduodenectomy may represent a major disadvantage. Perioperative pain management with IV-PCA may reduce the individual risk of postoperative complications. The PAKMAN trial will establish whether IV-PCA is able to improve patients’ surgical outcomes compared with EDA, thereby potentially changing medical practice.

## Methods/design

### Trial rationale

The aim of this pragmatic (effectiveness) trial is the comparison of two different perioperative pain-management strategies in pancreatic surgery with regard to gastrointestinal complications up to postoperative day (POD) 30. We postulate that the healing of anastomoses may be compromised by the increased intraoperative administration of vasopressors and intravenous fluids due to the vasodilatory effect of EDA. The primary objective of the trial is to investigate whether the frequency of the composite primary endpoint of gastrointestinal complications (delayed gastric emptying, pancreatic fistula, biliary leak, gastrointestinal bleeding, or postoperative ileus) following pancreatoduodenectomy could be reduced by 15 % by postoperative IV-PCA compared with thoracic EDA.

### Trial design

PAKMAN is an investigator-initiated, adaptive, pragmatic, randomized, controlled, multicenter, open-label, superiority trial with two parallel study groups.

### Eligibility

#### Eligibility criteria for participating trial centers

Established trial infrastructures from previously conducted multicenter trials (DISPACT and RecoPanc) will be used for the PAKMAN trial [3, 10]. Eight German (Dresden, Freiburg, Giessen, Heidelberg, Lübeck, Munich LMU, Stuttgart, and Tübingen), and three other European (Liverpool, Ljubljana, and Verona) high-volume centers with broad expertise in pancreatic surgery will be included.

#### Inclusion criteria

All patients scheduled for elective pancreatoduodenectomy at one of the participating centers will be screened consecutively for eligibility and will be informed about the PAKMAN trial during a pretreatment visit or on the day of admission to the hospital. All subjects must be able to understand the nature and potential individual consequences of the clinical trial, and only adult patients (≥ 18 years of age) who provide written informed consent will be included.

#### Exclusion criteria

Patients with chronic pancreatitis and patients with chronic pain syndrome for any reason will not be admitted to the PAKMAN trial because of possible pre-existing pain and tolerance to pain medication. Moreover, patients with severe chronic obstructive pulmonary disease (COPD, stage ≥ III according to the Global Initiative for Chronic Obstructive Lung Disease [GOLD] criteria) [11], or American Society of Anesthesiologists (ASA) physical status classification ≥ IV will be excluded. Furthermore, the presence of contraindication(s) to the use of IV-PCA or EDA (e.g., coagulopathies or allergies), participation in another intervention trial that interferes with the intervention or outcome of this trial, impaired mental state, or language problems will also prohibit inclusion in the study.

#### Subject withdrawal criteria

Subjects may be withdrawn from the trial at their own request or if a pancreatoduodenectomy is not performed (e.g., because of technical irresectability or metastatic disease). Withdrawn patients will be included in the final report of the trial to ensure complete transparency.

### Interventions

#### Pancreatoduodenectomy

The pancreatoduodenectomy may be performed as a classical, pylorus-preserving, or pylorus-resecting Whipple procedure. Furthermore, partial resection of the portal or superior mesenteric vein is allowed. Pancreatic anastomosis may be performed according to the local standard.

#### Description of trial interventions

All surgeons and anesthetists who participate in the PAKMAN trial will receive instructions on which treatment procedures are applicable to the two groups. Due to the nature of a pragmatic trial with only a few exclusion criteria and that focuses on effectiveness, no further efforts are proposed to standardize the applied interventions. In both groups, patients will receive intraoperative general anesthesia according to local practice.

#### Intervention group A (IV-PCA)

For postoperative analgesia, intravenous opioids will be used with the help of a patient-controlled-analgesia device according to local practice. The type of medication, dosage, and additional pain medication will be documented in detail in the CRF.

#### Intervention group B (EDA)

Prior to induction with general anesthesia, a thoracic epidural catheter will be inserted. This catheter will be used intraoperatively and postoperatively according to local standards in order to achieve anesthesia and analgesia, respectively. Therefore, local anesthetics will be injected with or without opioid analgesics into the epidural space. EDA has to be used postoperatively for at least 2 days. The type of medication, dosage, and additional pain medication will be documented in detail in the CRF.

### Concomitant treatment

All additional medications and/or treatments will be permitted during the trial when considered necessary by the treating physicians. In the event of failed EDA or premature cessation of the EDA, intravenous opioid analgesia will be given as a rescue medication and documented. If epidural hematoma or neurological complications occur, the standardized diagnostic and treatment algorithm suggested by Pöpping et al. will be used to assure patient safety [12]. The use of transverse abdominal plane blocks, wound infusion catheters, intrathecal medication, or perioperative IV infusion of lidocaine will be prohibited.

### Assignment of intervention and randomization

Consecutively screened and eligible patients will be included in the trial at each center after initiation of the study. To achieve comparable intervention groups for known and unknown risk factors, patients will be allocated in a concealed fashion by preoperative randomization 1 day before surgery using a centralized web-based tool (randomizer.at) provided by the Institute of Medical Informatics, Statistics, and Documentation of the Medical University of Graz, which has been successfully used in previous trials. Stratification by center will be performed to minimize center effects. Therefore, selection bias (biased allocation to interventions) will be minimized by sequence generation and allocation concealment. Randomization will be conducted exclusively by authorized trial personnel.

### Primary and secondary endpoints

#### Primary endpoint

The primary endpoint, defined according to the retrospective trial by Pratt et al. that was used for sample-size calculation [5], is the occurrence of at least one of the following gastrointestinal complications on or before POD 30:

- Delayed gastric emptying (grade A – C according to the ISGPS (International Study Group of Pancreatic Surgery) consensus definition) [13]
- Pancreatic fistula (grade A – C according to the ISGPF (International Study Group of Pancreatic Fistula) consensus definition) [14]
- Biliary leak (grade A – C according to the ISGLS (International Study Group of Liver Surgery) consensus definition) [15]
- Gastrointestinal bleeding (hematemesis, hematochezia, or melena and no other source of ongoing blood loss or the sudden appearance of clinically evident blood either on nasogastric lavage or per rectum, with a subsequent fall in hemoglobin of 2 mg/dl and requiring blood product transfusion, reoperation, or re-intervention (e.g., therapeutic endoscopy or angiography))
- Postoperative ileus (absence of bowel sounds, failure to pass flatus, or absence of bowel movement by POD 5)

The composite primary endpoint “gastrointestinal complications” will be assessed from the date of pancreatoduodenectomy up to the end of the study on POD 30. Outcome observers will assess the presence of the primary endpoint during clinical visits and from the patients’ files and by a telephone call on POD 30. If at least one item is assessed as present, the primary endpoint will have been reached for this patient. The severity of each complication will be classified by consensus and according to the Clavien-Dindo classification [16]. If no consensus definition can be reached, the severity will be assessed according to the Clavien-Dindo classification only.

#### Key secondary endpoints

Secondary endpoints (Table 1) will be assessed by authorized trial personnel during inpatient treatment and by means of a telephone visit after discharge of the patient. Single items of the composite primary endpoint will also be assessed as secondary endpoints. The complete listing of secondary endpoints can be found in Additional file 1.

**Table 1:** Definition and assessment of key secondary endpoints

Secondary endpoint	Definition	Assessment
Pneumonia	Presence of new infiltrate on chest x-ray OR CT scan	Yes/no (severity according to Clavien-Dindo classification)
Mortality	Death before POD 30 during hospital stay as well as after discharge	Yes/no, cause of death, and date of death
Hospital stay	Days from day of initial operation to day of hospital discharge	Days of inpatient treatment
Fluids given intraoperatively	Amount of fluids given intraoperatively	Crystalloid fluids in milliliters Colloidal fluids in milliliters
Vasopressor therapy intraoperatively	Amount of vasopressor during operation	Type of vasopressor, amount in milligrams
Fluids given postoperatively	Amount of fluids given postoperatively until POD 4 or death	Crystalloid fluids in milliliters, colloidal fluids in milliliters
Vasopressor therapy postoperatively	Amount of vasopressor after operation until POD 4 or death	Type of vasopressor, amount in milligrams
Weight over time/weight changes	Patient’s weight on day of screening, POD 2, and POD 4	Weight in kilograms
Re-operation	Reoperation up to POD 30 or death	Date and cause of every reoperation
Postoperative pain	Pain level on POD 2 and 4 during movement and at rest (NRS)	NRS

*NRS* numeric rating scale, *POD* postoperative day

### Description of trial visits

During the screening visit (between 1 week and 1 day before operation), the inclusion and exclusion criteria will be assessed. After the patient has given informed consent, the demographic and baseline data, medical history, current medication, comorbidities, prior imaging studies, ASA class, Karnofsky Index, and Charlson Comorbidity Index will be assessed and documented. Randomization will be performed on the day before surgery by authorized personnel only. On the day of surgery, visit 2 will be performed, with assessment of intraoperative and perioperative parameters as well as serious adverse events (SAEs). Patients will be followed up for 30 days after pancreatoduodenectomy, with follow-up visits scheduled on POD 2, 4, and 7 (POD 2 and 4 for pain and weight), and on day 14 or the day of discharge (whichever occurs first). During the postoperative visits, the primary and secondary endpoints, final histopathology, and SAEs will be assessed and documented. All planned study visits are summarized in Table 2. Data from patients without partial pancreatoduodenectomy (distal resection, total pancreatectomy, enucleation, or exploration only) will be documented up to the time of surgery (visit 2).

**Table 2:** Planned investigation scheme

Visit	V1	V2	V3	V4	V5	V6	V7
	Before surgery (days -7 to -1)	Day of surgery	POD 2	POD 4	POD 7	POD 14 or day of discharge	POD 30/EoS/premature termination
	Inpatient						Telephone
Inclusion/exclusion criteria and informed consent	X						
Demographic and clinical baseline data	X						
Randomization	X^a^						
Intervention		X					
Primary endpoint, assessment of gastrointestinal complications			X	X	X	X	X
Secondary endpoints		X	X	X	X	X	X
Pain (NRS)			X	X			
Weight			X	X			
SAE		X	X	X	X	X	X

*EoS* end of study, *V* visit, *POD* postoperative day, *SAE* serious adverse event, *NRS* numeric rating scale

^a^Randomization 1 day before operation

### Safety aspects

As the two interventions compared in this trial are both well-established and routinely used, only events fulfilling at least one of the criteria for SAEs have to be reported. SAEs occurring in the period from the day of randomization to the end of follow-up have to be documented and reported to the coordinating investigator within 5 days after they have become known. All ongoing SAEs (including SAEs in withdrawn subjects) have to be followed up until no more signs and symptoms are verifiable or the patient is in a stable condition. Planned elective admission to the hospital and planned elective surgery need not be reported because these SAEs are assumed unrelated to the trial intervention.

### Statistics

#### Hypotheses

The primary question is whether the rate of gastrointestinal complications is lower in one of the two intervention groups. Let *P*_1_ be the rate of complications in the IV-PCA group and *P*_2_ the rate of complications in the EDA group. The null hypothesis (H_0_: *P*_1_ = *P*_2_, no difference in the rate of gastrointestinal complications between the two intervention groups) is tested against the alternative hypothesis (H_1_: *P*_1_ ≠ *P*_2,_ different rates of gastrointestinal complications in the two groups).

This two-sided problem is tested with a logistic regression model, which will include the covariates center, age, body mass index, gender, and surgeon’s experience. Confirmatory analysis of the primary endpoint will be based on the full analysis set (FAS), which is consistent with the modified intention-to-treat (mITT) principle by the inclusion of every patient who was randomized to one of the study groups and received a pancreatoduodenectomy. This approach reflects the idea that the study results should match as closely as possible the conditions in clinical practice.

#### Sample size calculation

**Fig. 1:**
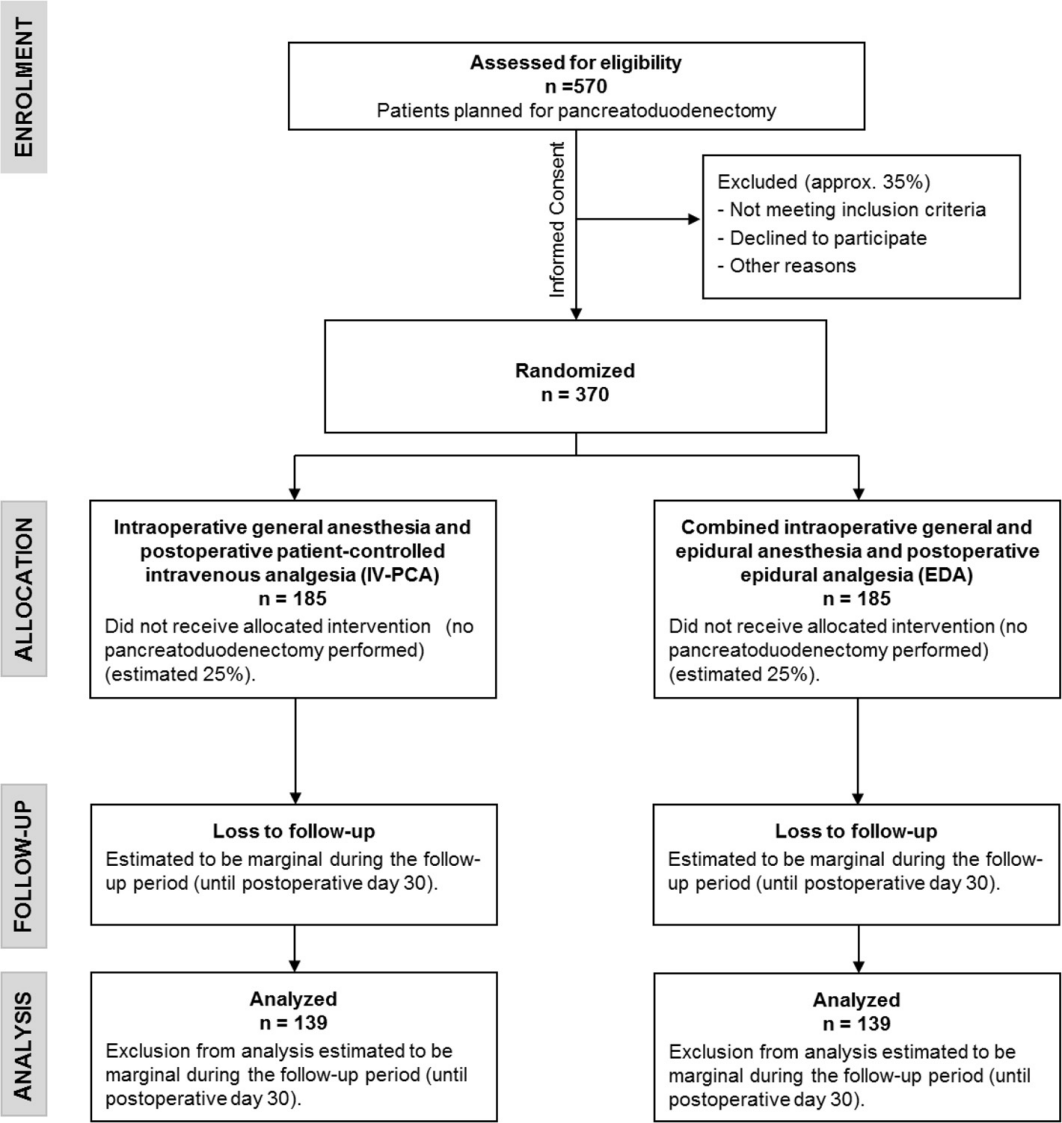
Planned investigation scheme

The sample size calculation is based on the difference in the frequency of gastrointestinal complications between postoperative IV-PCA and thoracic EDA up to POD 30, as it is very unlikely that any of the complications combined in the primary endpoint will occur later than 30 days after surgery. The prior assumption is based on the retrospective trial by Pratt et al. [5], who reported a 15 % difference (19 % versus 34 %) in the gastrointestinal complication rate up to POD 30. Due to the possible imprecision of the reported rates, a two-stage group sequential design according to O’Brien and Fleming [17] has been chosen, with the option of halting the trial for reasons of either futility or efficacy after the primary endpoints of two-thirds of the total patients are known.. The study will be stopped prematurely if the first-stage *p*-value lies below 0.0146 or above 0.4. With a level of significance *a* = 5 % and a power of 1-*b* = 80 %, a total sample size of 278 patients (139 per group) is sufficient when applying a two-sided chi-square test (average sample size under H_0_, 201.6; under H_1_, 228.9; calculated with ADDPLAN6). To account for the possibility that the observed difference may be diminished by patient noncompliance and/or dropout (assumed not to exceed 25 %), an additional 92 patients will be recruited to correct for these effects, resulting in 370 patients (Figure 1).

#### Interim analysis

As soon as the primary endpoint has been assessed in two-thirds (*n* = 185) of the total number of patients to be randomized, an interim analysis will be performed. If the study continues to the second stage, sample size adaptation will be applied according to the inverse normal method [17], thereby controlling the overall two-sided significance level of α = 0.05. Specific adaptation scenarios were considered with simulations (100,000 replications each). These included adaptive doubling, tripling, or quadrupling of the second-stage sample size after the interim analysis. The results showed that sufficient power would then be achieved, even if the relative treatment effect of the intervention were overestimated by 10 %, 15 %, or 18 %, respectively. However, when adaptations are performed, the adaptive designs do not apply sufficient test statistics and are less efficient than the fixed designs planned for this effect [18]. The amount of inefficiency was investigated and will be considered in the interim decision.

#### Premature closure of the trial

As described above, the trial will be stopped if the *p*-value resulting from the planned interim analysis lies below 0.0146 (for efficacy) or above 0.4 (for futility), to ensure the statistical validity of the applied two-stage group sequential design. It will also be stopped if the interim analysis permits continuation of the study but the recalculated sample size to achieve sufficient statistical power is infeasible or inefficiently high.

#### Further analyses

An additional evaluation (sensitivity analysis) will be performed with the per protocol set, and the results will be compared with those of the ITT analysis. If a patient leaves the trial prematurely without having experienced an event with respect to the primary endpoint, the missing data for the primary outcome variable will be replaced using the ICA-r method described by Higgins et al. [19]. Concerning secondary endpoints, exploratory data analysis will be performed, with both appropriate summary measures for the empirical distribution and descriptive two-sided *p*-values being calculated. The safety analysis includes the calculation and comparison of frequencies and rates of serious SAEs. Furthermore, statistical methods are used to assess the quality of data and the homogeneity of the intervention groups. Details of the statistical analysis will be fixed at the latest in the statistical analysis plan to be prepared before database lock and commencement of analysis. All analyses will be conducted using SAS 9.1 or higher.

#### Data management

All protocol-required information collected during the trial must be entered into the case report form (CRF) by the investigator or a designated representative. The investigator or a designated representative must complete the CRF as soon as possible after the information is collected, preferably on the same day that a trial subject is seen for an examination, treatment, or any other trial procedure. Any outstanding entries must be completed immediately after the final examination. An explanation must be given for any missing data.

The completed CRF must be reviewed and signed by the investigator or by a designated subinvestigator. A copy will be retained at the trial center, and the original CRF will be sent to the Institute of Medical Biometry and Informatics Heidelberg (IMBI), which is in charge of data management for the PAKMAN trial.

To ensure that the database reproduces the CRFs correctly, the IMBI will perform the data entry twice. IMBI representatives will check completeness, validity, and plausibility of all data using validation programs, which will generate queries. All validation rules will be predefined in a data-validation plan. The investigator, or a designated representative, will be obliged to resolve the queries. As soon as no further corrections or clarifications are required, the database will be closed and used for statistical analysis. The data will be managed and analyzed according to the appropriate standard operating procedures of the IMBI.

#### Data monitoring

Monitoring will be conducted according to approved standard operating procedures, which include personal onsite visits with source data verification. Clinical monitoring within the PAKMAN trial will be performed according to the ICH-GCP guideline (E6) by the Coordination Centre for Clinical Trials (KKS) Heidelberg. Monitoring procedures will be predefined in a study-specific monitoring manual. Monitoring details will be adapted to the study-specific risks for the patients.

### Ethical and legal aspects

The responsible investigator will ensure that the PAKMAN trial adheres to the tenets of the Declaration of Helsinki in its current version and to the laws and regulations of the country concerned. This protocol is designed to ensure that the trial will be conducted and analyzed in accordance with ICH E6 Good Clinical Practice [20]. The protocol has already been approved by the independent ethics committee (IEC) of the medical faculty of the University of Heidelberg, and secondary approval of the corresponding ethical bodies of all other participating centers has been or will be obtained. Additional file 2 contains a list of all local ethical bodies that have approved the PAKMAN trial. Recruitment in any center will not start before its individual ethical approval has been obtained. Any amendments will be signed by all parties and submitted to all of the IEC, and the IEC will also be informed at the end of the trial. The trial protocol has been formulated in accordance with the recommendations of the CONSORT and Standard Protocol Items: Recommendations for Interventional Trials (SPIRIT) guidelines (Additional file 2) [21, 22].

## Discussion

Previous research comparing IV-PCA and EDA has focused mainly on the level of pain control itself [4, 23] or on the incidence of cardiac and pulmonary complications [4, 6]. The reported mean visual analog pain scores are 3.2 ± 1.6 with IV-PCA versus 2.1 ± 1.3 with EDA. This difference is statistically significant but of minor clinical relevance at best and diminishes during the postoperative course [4, 23]. In addition, up to 30 % of the patients with EDA still experience severe pain in clinical practice [24]. In contrast to the subjective belief of many anesthetists, the failure of epidural anesthesia and analgesia is a frequent clinical problem (failure rate 13 % to 47 % in experienced hands) [23, 25].

The postulated benefits of EDA are reduced consumption of anesthetics, improved pain control with higher patient satisfaction, reduced incidence of cardiac and pulmonary complications, reduction of the surgical stress response, early bowel recovery, and enhanced postoperative recovery with shorter hospital stay [4, 6]. Despite these postulated benefits, the influence of EDA on clinically relevant outcomes is not clear [26]. Although conclusions from historical studies have been promising, recent studies have shown no relevant effect [23]. Only weak evidence exists that EDA is able to decrease postoperative cardiovascular and pulmonary complications for subgroups with major open vascular surgery or high-risk patients [23, 27]. A 2011 meta-analysis investigating the effects of thoracic EDA on perioperative outcome failed to show a benefit on perioperative in-hospital mortality in noncardiac surgery [7]. In a single-center RCT, the preemptive use of EDA in patients undergoing open colon resection offered no advantage over IV-PCA with regard to the length of the hospital stay, pain scores, quality of life, complications, or hospital costs [28]. Even in high-risk patients undergoing major abdominal surgery, most adverse outcomes are not reduced by the use of EDA [29].

Furthermore, the risk of epidural hematoma and paraplegia after EDA seems to be higher than previously assumed [12]. The German network for safety in regional anesthesia reported the incidence of epidural hematoma after EDA as 1:6,628 [30].

Pain control and the incidence of cardiac or pulmonary complications are critical to patients but form only a minor part of patient-relevant outcome parameters after major surgical operations such as pancreatoduodenectomy. Most trials to date have not correlated analgesic treatment with surgical complications. Our primary endpoint is of high clinical relevance for patients undergoing pancreatoduodenectomy because nearly every single gastrointestinal complication easily meets the definition of an SAE, and the high morbidity is mainly caused by these very complications. The validity and reproducibility of our results will be secured by a clear-cut definition of all outcome parameters and the use of international consensus definitions, when available.

The sample size calculation is based on a single retrospective study [5], and possibly, the rates reported there are imprecise. For this reason, a two-stage group sequential design is planned, with the option of stopping both for futility and for efficacy after the primary endpoint is reached in two-thirds of the total patients. In the case of continuation of the trial, the sample size will be adjusted. Furthermore, to account for possible noncompliance and dropout of patients, 25 % more participants will be recruited than are needed.

The PAKMAN trial is designed as a pragmatic trial because the objective is to depict the effectiveness of interventions in real-life routine practice conditions and produce results that can be generalized. The broad inclusion and narrow exclusion criteria reflect the pragmatic nature of the trial and the aim to obtain results with high generalizability and high external validity. Therefore, a number of interference factors, such as the experience of the surgeon (in terms of number of pancreatoduodenectomies performed (< 26, 26-50, 51-75, or > 75)), the underlying disease (benign versus malignant), the method of pancreatic anastomosis (pancreatogastrostomy versus pancreatojejunostomy), and the type of general anesthesia (medication, dosage, and type of application) will be assessed and available for subgroup analysis. These factors, however, were not predetermined in the study protocol. Moreover, by randomizing 370 patients, the known and unknown confounders will be distributed equally.

The risk of performance bias due to nonblinding is low because objective endpoints are used. Blinding of the patients and treating physicians is not possible as a matter of principle. The impact of a blinded observer on the reduction of bias would only be marginal because the primary endpoint (delayed gastric emptying, pancreatic fistula, biliary leak, gastrointestinal bleeding, or postoperative ileus) is defined in detail and already well documented (e.g., operation or intervention reports) in daily practice. It seems unlikely that these objective trial results will differ significantly if the observer is unblinded [31].

The difference on which the PAKMAN trial focuses is a qualitative one (induced sympathicolysis due to any kind of EDA against IV-PCA without sympathicolysis). Therefore, only particular key steps of the interventions are predefined, and procedures can otherwise be performed according to local practice. EDA and IV-PCA medication, dose, and mode of application will be documented but not standardized. EDA and IV-PCA will be performed according to the centers’ standard procedures. Nevertheless, the detailed documentation will allow additional subgroup analysis regarding type/dose of medication for EDA or IV-PCA and frequency of gastrointestinal complications.

The results of the PAKMAN trial could influence future guidelines and decision-making. Healthcare costs might be decreased by a reduction of the complications themselves and of the treatment and hospital stay required to deal with the complications. Furthermore, any other treatment needed by patients (e.g., chemotherapy) might be delayed less often by complications.

### Trial status

Recruitment of patients for the PAKMAN trial started in June 2015 (the first patient was randomized on 30 June 2015), and the trial is expected to be complete in 2017.

## Additional files

Additional file 1: Definition and assessment of all secondary endpoints. (DOC 50 kb) Additional file 2: Names of all local ethical bodies that have approved the PAKMAN trial. (DOC 22 kb)

## Abbreviations

ASA: American Society of Anesthesiologists
COPD: chronic obstructive pulmonary disease
CRF: case report form
CT: computer tomography
DRKS: *Deutsches Register klinische Studien* (German Clinical Trials Register)
EDA: epidural analgesia
EoS: end of study
FAS: full analysis set
FFP: fresh frozen plasma
GCP: good clinical practice
GOLD: Global Initiative for Chronic Obstructive Lung Disease
ICH: International Conference on Harmonisation of Technical Requirements for Registration of Pharmaceuticals for Human Use
IEC: independent ethics committee
IMBI: Institute of Medical Biometry and Informatics Heidelberg
ISGLS: International Study Group of Liver Surgery
ISGPF: International Study Group on Pancreatic Fistula
ISGPS: International Study Group on Pancreatic Surgery
ITT: intention to treat
IV-PCA: intravenous patient-controlled analgesia
LPO: last patient out
mITT: modified intention to treat
MRI: magnetic resonance imaging
NRS: numeric rating scale
PCA: patient-controlled analgesia
POD: postoperative day
RCT: randomized controlled trial
SAE: serious adverse event
V: visit
WBC: white blood cells

## References

[1] Winter JM, Cameron JL, Campbell KA, Arnold MA, Chang DC, Coleman J (2006). 1423 pancreaticoduodenectomies for pancreatic cancer: a single-institution experience. J Gastrointest Surg.

[2] Crippa S, Salvia R, Falconi M, Butturini G, Landoni L, Bassi C (2007). Anastomotic leakage in pancreatic surgery. HPB (Oxford).

[3] Keck T, Wellner U, Bahra M, Klein F, Sick O, Niedergethmann M, et al. Pancreatogastrostomy versus pancreatojejunostomy for reconstruction after pancreatoduodenectomy (RECOPANC, DRKS 00000767): perioperative and long-term results of a multicenter randomized controlled trial. Ann Surg. 2015. in press. 10.1097/SLA.0000000000001240 PMC4741417 26135690

[4] Wu CL, Cohen SR, Richman JM, Rowlingson AJ, Courpas GE, Cheung K (2005). Efficacy of postoperative patient-controlled and continuous infusion epidural analgesia versus intravenous patient-controlled analgesia with opioids: a meta-analysis. Anesthesiology.

[5] Pratt WB, Steinbrook RA, Maithel SK, Vanounou T, Callery MP, Vollmer CM (2008). Epidural analgesia for pancreatoduodenectomy: a critical appraisal. J Gastrointest Surg.

[6] Panousis P, Heller AR, Koch T, Litz RJ (2009). Epidural ropivacaine concentrations for intraoperative analgesia during major upper abdominal surgery: a prospective, randomized, double-blinded, placebo-controlled study. Anesth Analg.

[7] Gauss PDA, Jahn S, Eberhart L, Stahl W, Rockemann M, Georgieff M (2011). Kardioprotektion durch thorakale periduralanästhesie?. Anaesthesist.

[8] Tassoudis V, Vretzakis G, Petsiti A, Stamatiou G, Bouzia K, Melekos M (2011). Impact of intraoperative hypotension on hospital stay in major abdominal surgery. J Anesth.

[9] Fischer M, Matsuo K, Gonen M, Grant F, DeMatteo RP, D’Angelica MI (2010). Relationship between intraoperative fluid administration and perioperative outcome after pancreaticoduodenectomy: results of a prospective randomized trial of acute normovolemic hemodilution compared with standard intraoperative management. Ann Surg.

[10] Diener MK, Seiler CM, Rossion I, Kleeff J, Glanemann M, Butturini G (2011). Efficacy of stapler versus hand-sewn closure after distal pancreatectomy (DISPACT): a randomised, controlled multicentre trial. Lancet.

[11] Pauwels RA, Buist AS, Calverley PMA, Jenkins CR, Hurd SS. Global strategy for the diagnosis, management, and prevention of chronic obstructive pulmonary disease. Am J Resp Crit Care Med. 2001;163(5):1256–76. 10.1164/ajrccm.163.5.2101039 11316667

[12] Pöpping DM, Wenk M, Van Aken HK (2012). Neurologic complications after epidural analgesia. Anasth Intensiv Notf.

[13] Wente MN, Bassi C, Dervenis C, Fingerhut A, Gouma DJ, Izbicki JR (2007). Delayed gastric emptying (DGE) after pancreatic surgery: a suggested definition by the International Study Group of Pancreatic Surgery (ISGPS). Surgery.

[14] Bassi C, Dervenis C, Butturini G, Fingerhut A, Yeo C, Izbicki J (2005). Postoperative pancreatic fistula: an international study group (ISGPF) definition. Surgery.

[15] Koch M, Garden OJ, Padbury R, Rahbari NN, Adam R, Capussotti L (2011). Bile leakage after hepatobiliary and pancreatic surgery: a definition and grading of severity by the International Study Group of Liver Surgery. Surgery.

[16] Dindo D, Demartines N, Clavien P-A (2004). Classification of surgical complications: a new proposal with evaluation in a cohort of 6336 patients and results of a survey. Ann Surg.

[17] O’Brien PC, Fleming TR. A multiple testing procedure for clinical trials. Biometrics. 1979;549–56. 497341

[18] Tsiatis AA, Mehta C (2003). On the inefficiency of the adaptive design for monitoring clinical trials. Biometrika.

[19] Higgins JP, White IR, Wood AM (2008). Imputation methods for missing outcome data in meta-analysis of clinical trials. Clinical Trials.

[20] IHT. Guideline for Good Clinical Practice E6 (R1). Current Step 4 version dated 10 June 1996. http://www.ich.org/products/guidelines/efficacy/efficacy-single/article/good-clinical-practice.html

[21] Moher D, Hopewell S, Schulz KF, Montori V, Gøtzsche PC, Devereaux P (2010). CONSORT 2010 explanation and elaboration: updated guidelines for reporting parallel group randomised trials. J Clin Epidemiol.

[22] Chan A-W, Tetzlaff JM, Gøtzsche PC, Altman DG, Mann H, Berlin JA (2013). SPIRIT 2013 explanation and elaboration: guidance for protocols of clinical trials. BMJ..

[23] Kooij FO, Schlack WS, Preckel B, Hollmann MW (2014). Does regional analgesia for major surgery improve outcome? Focus on epidural analgesia. Anesth Analg.

[24] Duncan F (2011). Prospective observational study of postoperative epidural analgesia for major abdominal surgery. J Clin Nurs.

[25] Hermanides J, Hollmann M, Stevens M, Lirk P (2012). Failed epidural: causes and management. Brit J Anaesth.

[26] Gendall K, Kennedy R, Watson A, Frizelle F (2007). The effect of epidural analgesia on postoperative outcome after colorectal surgery. Colorectal Dis.

[27] Liu SS, Wu CL (2007). Effect of postoperative analgesia on major postoperative complications: a systematic update of the evidence. Anesth Analg.

[28] Zutshi M, Delaney CP, Senagore AJ, Mekhail N, Lewis B, Connor JT (2005). Randomized controlled trial comparing the controlled rehabilitation with early ambulation and diet pathway versus the controlled rehabilitation with early ambulation and diet with preemptive epidural anesthesia/analgesia after laparotomy and intestinal resection. Am J Surg.

[29] Rigg JR, Jamrozik K, Myles PS, Silbert BS, Peyton PJ, Parsons RW (2002). Epidural anaesthesia and analgesia and outcome of major surgery: a randomised trial. Lancet.

[30] Volk T, Wolf A, Van Aken H, Bürkle H, Wiebalck A, Steinfeldt T (2012). Incidence of spinal haematoma after epidural puncture: analysis from the German network for safety in regional anaesthesia. Eur J Anaesth (EJA).

[31] Higgins JP (2008). Green S.

