# Supplementary material for: Intravenous versus epidural analgesia to reduce the incidence of gastrointestinal complications after elective pancreatoduodenectomy (the PAKMAN trial, DRKS 00007784): study protocol for a randomized controlled trial
Source: Trials. 2016 Apr 11;17:194. doi: 10.1186/s13063-016-1306-4 (PMC4827246; doi:10.1186/s13063-016-1306-4)
Supplement: Additional file 1: — Definition and assessment of all secondary endpoints. (DOC 50 kb) [file 13063_2016_1306_MOESM1_ESM.doc]

Additional file 1: Definition and assessment of all secondary endpoints

| **Secondary endpoint** | **Definition** | **Assessment** |
| --- | --- | --- |
| Delayed gastric emptying | ISGPS consensus definition | Yes/no (severity according to Clavien-Dindo classification and Grade A – C) |
| Pancreatic fistula | ISGPF consensus definition | Yes/no (severity according to Clavien-Dindo classification and Grade A – C) |
| Biliary leakage | ISGLS consensus definition | Yes/no (severity according to Clavien-Dindo classification and Grade A – C) |
| Gastrointestinal bleeding | Hematemesis, hematochezia, or melena and no other source of ongoing blood loss or the sudden appearance of clinically evident blood either on nasogastric lavage or per rectum, with subsequent fall in hemoglobin of 2 mg/dl, and requiring blood product transfusion, reoperation, or reintervention (e.g. therapeutic endoscopy or angiography). | Yes/no (severity according to Clavien-Dindo classification) |
| Ileus | Absence of bowel sound, failure to pass flatus or absence of bowel movement by POD 5 | Yes/no (severity according to Clavien-Dindo classification) |
| Neuroaxial hematoma | Bleeding into the vertebral canal confirmed by MRI or CT scan | Yes/no (severity according to Clavien-Dindo classification) |
| Neurological complication | New or progressive postoperative neurologic deficits, defined as the presence of motor deficits, sensory deficits, painful paresthesia, dysesthesias, or hyperreflexia. This also includes infectious complications such as epidural abscess, meningitis confirmed by MRI or CT scan or microbiological examinations | Yes/no (severity according to Clavien-Dindo classification) |
| Pneumonia | Presence of new infiltrate on chest x-ray or CT scan | Yes/no (severity according to Clavien-Dindo classification) |
| Urinary tract infection | Culture-positive urine or pyuria and bacteriuria on urinalysis requiring antibiotic treatment | Yes/no (severity according to Clavien-Dindo classification) |
| Wound infection | Superficial and deep surgical site infection according to Centers of disease control [27] | Yes/no (superficial/deep SSI, severity according to Clavien-Dindo classification) |
| Intra-abdominal abscess | Culture-positive purulent drainage from intra-abdominal fluid collection obtained percutaneously or operatively or radiographically confirmed fluid collection with systemic OR localized signs of infection (i.e. elevated WBC, body temperature >38°C, purulent drainage) | Yes/no (severity according to Clavien-Dindo classification) |
| Sepsis | Systemic inflammatory response syndrome with infectious origin | Yes/no (severity according to Clavien-Dindo classification) |
| Mortality | Death until POD 30 during hospital stay as well as after discharge | Yes/no, cause of death, and date of death |
| Hospital stay | Days from day of the initial operation to day of hospital discharge | Days of inpatient treatment |
| Intensive/ intermediate care unit stay | Treatment in intensive/ intermediate care setting on or after POD 1 to discharge from hospital | Days in intensive care unit |
| Readmission to hospital | Readmission for management of postoperative complications until POD 30 | Yes/no, date of readmission |
| Postoperative intraabdominal bleeding | ISGPS consensus definition for postpancreatectomy hemorrhage | Yes/no (severity according to Clavien-Dindo classification, grade A – C) |
| Operation time | Time from incision to skin closure | time (hh:mm) of incision to time (hh:mm) of skin closure |
| Need for blood products intraoperative | Units and volume of packed red blood cells during operation | Amount in ml |
|  | Units and volume of FFP cells during operation | Amount in ml |
|  | Units and volume of thrombocytes during operation | Amount in ml |
| Fluids given intraoperatively | Amount of fluids given intraoperatively | Crystalloid fluids in ml  Colloidal fluids in ml |
| Need for blood products postoperatively until POD 4 | Units/amount of packed red blood cells | Amount in ml |
|  | Units/amount of FFP | Amount in ml |
|  | Units/amount of thrombocytes | Amount in ml |
| Vasopressor therapy intraoperatively | Amount of vasopressor during operation | Type of vasopressor,  amount in mg |
| Fluids given postoperatively | Amount of fluids given postoperatively until POD 4 or death | Crystalloid fluids in ml,  colloidal fluids in ml |
| Vasopressor therapy postoperatively | Amount of vasopressor after operation until POD 4 or death | Type of vasopressor,  amount in mg |
| Weight over time/weight changes | Patient’s weight on day of screening, POD 2, and POD 4 | Weight in kilogram |
| Reoperation | Reoperation up to POD 30 or death | Date and cause of every reoperation |
| Postoperative pain | Pain level on POD 2 and 4 during movement and at rest (NRS) | NRS |

ISGPS: International study group on pancreatic surgery; ISGPF: International study group on pancreatic fistula; ISGLS: International study group of liver surgery; POD: postoperative day; MRI: magnetic resonance imaging; CT: computer tomography; WBC: white blood cells; FFP: fresh frozen plasma; NRS: numeric rating scale
