# Supplementary material for: Intravenous versus epidural analgesia to reduce the incidence of gastrointestinal complications after elective pancreatoduodenectomy (the PAKMAN trial, DRKS 00007784): study protocol for a randomized controlled trial
Source: Trials. 2016 Apr 11;17:194. doi: 10.1186/s13063-016-1306-4 (PMC4827246; doi:10.1186/s13063-016-1306-4)
Supplement: Additional file 2: — Names of all local ethical bodies that have approved the PAKMAN trial. (DOC 22 kb) [file 13063_2016_1306_MOESM2_ESM.doc]

Additional file 2: Names of all local ethical bodies that have approved the PAKMAN trial

Ethikkommission des Universitätsklinikums Freiburg (Ethics-Commission of the Medical Center – University of Freiburg)

Ethikkommission am Fachbereich Medizin Universität Gießen (Ethics Committee at the Medical Faculty of the University of Giessen)

Ethik-Kommission I der Medizinischen Fakultät Heidelberg (Ethics Committee of the Medical Faculty of the University of heidelberg)

Ethikkommission der Universität Lübeck (Ethics Committee of the University of Lübeck)

Ethikkommission der Landesärztekammer Baden-Württemberg (Ethics Committee of the State Chamber of Physicians Baden-Wuerttemberg)

Ethikkommission bei der Medizinischen Fakultät Universität Würzburg (Ethics Committee at the Medical Faculty University of Wuerzburg
